# The Art of Safe and Judicious Deprescribing in an Elderly Patient: A Case Report

**DOI:** 10.3390/geriatrics5030057

**Published:** 2020-09-21

**Authors:** Pietro Gareri, Luca Gallelli, Antonino Maria Cotroneo, Valeria Graziella Laura Manfredi, Giovambattista De Sarro

**Affiliations:** 1 Department of Primary Care–Azienda Sanitaria Provinciale, Center for Cognitive Disorders and Dementia, 88100 Catanzaro, Italy; pietro.gareri@alice.it; 2 Department of Health Science, School of Medicine, University “Magna Graecia” Catanzaro, 88100 Catanzaro, Italy; desarro@unicz.it; 3 Operative Unit of Clinical Pharmacology, University Hospital of Mater Domini Catanzaro, 88100 Catanzaro, Italy; 4 Hospital Maria Vittoria and Territorial Geriatrics Botticelli, 10123 Turin, Italy; geriatrix1@libero.it; 5 S. Anna Hospital, 88100 Catanzaro, Italy; valeriagl.manfredi@hotmail.it

**Keywords:** elderly, polypharmacy, drug interactions, cognitive impairment, case report

## Abstract

Prescription for inappropriate drugs can be dangerous to the elderly due to the increased risk of adverse drug reactions and drug-interactions. In this manuscript, we report the complexity of polypharmacy and the possible harmful consequences in an old person. An 81-year-old man with a clinical history of diabetes, blood hypertension, non-valvular atrial fibrillation, chronic obstructive pulmonary disease, osteoarthritis, anxiety, and depression, was admitted to our attention for cognitive disorders and dementia. Brain magnetic resonance imaging showed parenchymal atrophy with lacunar state involving thalami and internal capsules. Neuropsychological tests revealed cognitive impairment and a depressed mood. History revealed that he was taking 11 different drug severy day with a potential risk of 55 drug–drug interactions. Therefore, risperidone, chlorpromazine, *N*-demethyl-diazepam, and L-DOPA/carbidopa were gradually discontinued and citicoline (1g/day), cholecalciferol (50,000 IU once a week), and escitalopram (5 mg/day) were started. Furthermore, he started a program of home rehabilitation. During the follow-up, three months later, we recorded an improvement in both mood and cognitive tests, as well as in walking ability. The present case report shows the need for a wise prescription and deprescribing in older people.

## 1. Introduction

Prescriptions for inappropriate drugs can be dangerous in older people, due to the development of adverse drug reactions and drug interactions [1,2,3,4,5,6,7].

Furthermore, age, gender, education, and, in general, sociodemographic factors, play an important role in drug use and abuse [8,9].

In particular, all the necessary practices for reducing the risk of inappropriate drug prescriptions should be the bread and butter for geriatricians, as well as for clinical pharmacologists [3,4].

A potentially inappropriate medication (PIM) is a drug that potentially increases the risk for adverse events when there is a safer alternative available [10].

The “Beers criteria” in older adults is the most validated tools for preventing PIM; they were started in 1991 and repeatedly revised, the last revision occurring in 2019 by the American Geriatric Society [11].

In this study, we report the complexity of poly-treatment and the possible harmful consequences in an older man.

## 2. Case Presentation

An 81-year old man lives with his 72-year old wife in a mountain town. He worked as a car mechanic and retired when he was 65 years old. He is right-handed and attended primary school. He never smoked, drinks a glass of wine at meals, and has a balanced diet. A couple of years prior, he complained of cognitive deficits, that were reported by his wife and one of his sons, such as difficulty in remembering appointments with friends, repetitiveness in his statements, loss of attention and concentration, anxiety and depressed mood, sporadic psychomotor restlessness, morning somnolence, and severe insomnia, with difficulty in both getting to sleep and early nocturnal awakenings. The patient suffered from hypertension (2004), diabetes (2007), and COPD (2008). Moreover, he was hospitalized in 2010 for acute coronary syndrome (ACS STEMI), treated with percutaneous coronary intervention (PCI) and stent placement on the left anterior descending artery, and in December 2017 and March 2018 for a transient ischemic attack. In 2019, a new clinical evaluation diagnosed a non-valvular atrial fibrillation in the patient with osteoarthritis and walking deficits, anxiety, mood depression, hypertension, and atrial fibrillation. Therefore, treatment with apixaban, bisoprolol, antianxiety drugs, and antidepressants was started (see Table 1). In February 2020, he was admitted to our evaluation for cognitive impairment and dementia; laboratory tests revealed the presence of hyperglycemia and low serum vitamin D levels (Table 2).

**Figure 1:**
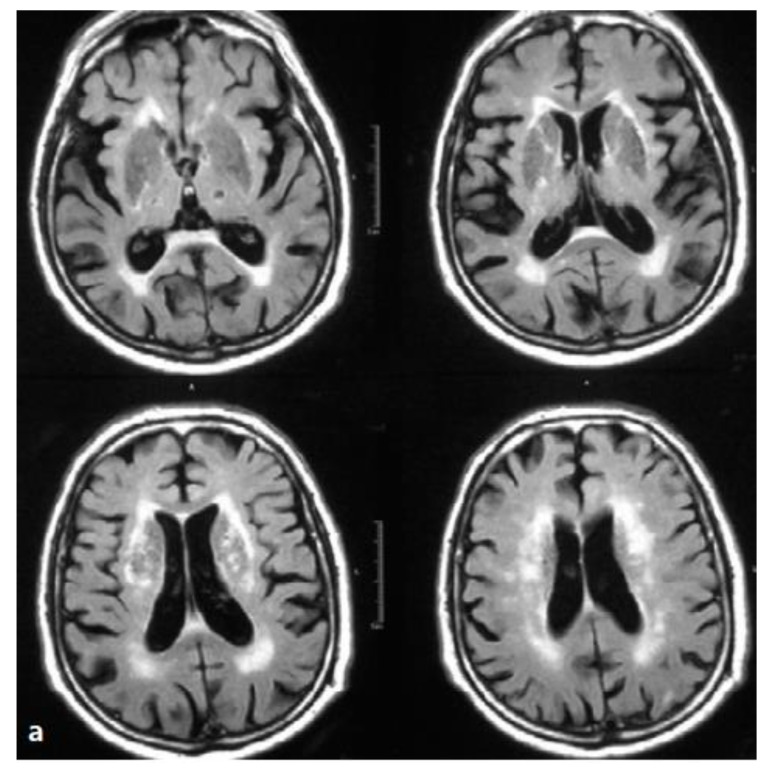
Brain MRI. Axial T2-weighted scans and fluid attenuation inversion recovery (FLAIR). Hypertension and recurrent *strokes* with subcortical lesions. Wide alteration of periventricular and lobar white matter signal. The image shows lacunar lesions involving thalami and internal capsules (lacunar state), together with the so-called “cribrose state”, with marked dilatation of base nuclei perivascular spaces.

A brain MRI scan showed parenchymal atrophy with lacunar state involving thalami and internal capsules (Figure 1).

Neuropsychological tests (Table 3) revealed a cognitive impairment, i.e., Mini Mental State Examination score 20.4/30. The Montreal Cognitive Assessment documented low values of 19/30. During the language tests, the repetition of a sentence was disordered, due to decreased attention and the patient named only seven words starting with the letter “F”. Moreover, all the other tests documented very low results, i.e., clock drawing test 6/10; Rey words 21 (cut-off 28.53); he committed two mistakes on the Stroop test, time 43 s; and did not manage to perform the Trail Making Test part B; Neuropsychiatric Inventory score was 27/144, with episodes of anxiety, occasional visual hallucinations, mild agitation and irritability; and Geriatric Depression Scale-15 items was 8/15, insight was maintained. Finally, we evaluated both the Activities of Daily Living and the Instrumental Activities of Daily Living and we obtained a score of 2/6 and 1/5, respectively (Table 3).

On physical examination, the patient presented heart frequency of 74 bpm, proto-meso-systolic murmur, hypertonus of probable iatrogenic origin (due to treatment with risperidone and chlorpromazine). Vesicular murmur was normal, abdomen was soft on palpation, and peripheral pulses were normal. Blood pressure was 130/80 mm Hg. Electrocardiography confirmed the presence of atrial fibrillation with heart frequency of 74 bpm. Echocardiography documented a normal ventricular activity (ejection fraction 55%).

After these evaluations, the patient was advised to stop treatment with risperidone, chlorpromazine, *N*-demethyl-diazepam (slow titration), and L-DOPA/carbidopa. Pantoprazole was stopped, but due to the presence of gastric pyrosis, magaldrate 80 mg at bedtime was added.

Finally, a treatment with citicoline (1g/day), cholecalciferol (50,000 IU once a week for three months after meals), and escitalopram (5 mg after breakfast) was started. The inappropriate drugs according to the Beers criteria are reported in Table 4.

Furthermore, the patient started a program of home rehabilitation. Three months later, during the follow-up, we documented an improvement in both mood and cognitive tests (in particular in orientation and in attention, MMSE (24.4/30, normal values 26–30), as well as in walking ability. A decrease in hypertonus was also recorded. ADL and IADL improved one score for each one (Table 3). The benefits were kept even after six months (Table 3).

This study was approved by local ethic committee (protocol number 29 of the 11 February 2016) and the patient give us the written informed consent for the publication.

The questions which arise due to these changes are the following:

1. Could the difficulty in planning and attention, as well as concentration deficits be explained by this kind of treatment?
2. Could the dramatic improvement recorded in our patient be due to changes in the therapeutic regimen?

The answer to both questions is yes, it could.

In particular, if we carefully examine the whole treatment which the patient underwent, we need to take into account that he was taking 11 medications distributed in 14 daily administrations. First of all, facing the challenge of polyprescriptions, it is well known among geriatricians and pharmacologist that changes in pharmacokinetics and pharmacodynamics are one of the pitfalls during aging [10]. Another important concern regards the possible drug–drug, drug–herbal products, drug–disease, and drug-over-the-counter drugs interactions [12]. The patient took 11 drugs, which means 55 potential interactions (number of interactions = (drug count) × (drug count − 1)/2).

To avoid potential inappropriate drug prescriptions, a number of criteria can be used; among them, the STOPP (Screening Tool of Older Persons’ Potentially Inappropriate Prescriptions) and START (Screening Tool to Alert Prescribers to Right Treatment) criteria [13].

The drugs that deserve the utmost attention are chlorpromazine, risperidone, levodopa/carbidopa, *N*-demethyl-diazepam, and pantoprazole.

Regarding chlorpromazine and risperidone, antipsychotics potentially increase the risk of sudden death, cerebrovascular events, as well as metabolic and hematological side effects [14]. They require monitoring of electrocardiogram’s QTc interval and serum electrolytes. They should be used for severe, persistent, and resistant aggression for a short time (6–12 weeks), and then their dosage needs to be reduced 25–50% every 1–2 weeks until stopping them [15]. Moreover, there is no reason for prescribing two antipsychotics such as chlorpromazine and risperidone; they can cause an increase in extrapyramidal side effects (EPS), especially in elderly people [16]. Another mistake is the use of L-DOPA for contrasting antipsychoticonset of EPS; antipsychotics, especially risperidone, together with L-DOPA are responsible for the so-called cascade prescription and lead to a pharmacodynamic antagonism.

*N*-demethyl-diazepam is a long half-life benzodiazepine, potentially leading to side effects and adverse events, such as paradoxical reactions, sedation, fractures, and cognitive worsening, or they can even increase the risk of Alzheimer’s disease [17]. Gradual and slow titration of *N*-demethyl-diazepam has been associated with improvement of cognition. In any case, short acting benzodiazepines should also be used for the shortest time possible (it is often said, “to start with the end in mind”).

Pantoprazole is a proton pump inhibitor (PPI) which is widely used for too long. It can induce headache, nausea, diarrhea and rash [18]. Furthermore, its use seems to be associated with a higher risk of fractures, hypomagnesemia, Clostridium difficile infections, community-acquired pneumonia, and vitamin B12 deficiency [18]; moreover, its chronic use has been associated with an increased risk of death for acute renal failure and cardiovascular disease [19]. Therefore, we also decided to discontinue pantoprazole.

Nevertheless, we must highlight the importance of the use of a cholinergic precursor such as citicoline, also called CDP-choline (cytidine-5′-diphosphate choline). Citicoline is used for cognitive impairment of different etiology, it has anti-apoptotic properties, especially inhibiting brain ischemia associated apoptosis [20]. Citicoline increases acetylcholine, dopamine, and noradrenaline intra-synaptic levels, and stimulates phospholipid synthesis (phosphatidylcholine) [20,21]. It has been shown to promote SIRT-1 activation, a neuroprotective protein, and promote both cell function and neuronal repair stimulation [20,22]. Citicoline was also been shown to be effective in mild vascular cognitive impairment and in Alzheimer’s disease, and mixed dementia in combination with acetylcholinesterase drugs or memantine [23,24,25,26]. Its efficacy is superior in long-term use (6–9 months and beyond) and this is one reason why, until now, this drug was not been used at its best [18]. The effects on cognition, mood and walking ability should be related to the increase in the intra-synaptic levels of neurotransmitters (acetylcholine, dopamine, noradrenaline, and, indirectly, serotonin).

Importantly, we also recorded a change in both ADL and IADL scores (improvement of one score for each one), due an improvement of functional abilities. This improvement was probably related to reduced stiffness, both for antipsychotics interruption and for home rehabilitation.

Finally, it is important to underline that this manuscript has some limitations related with the type of study. In particular, it is a case report, therefore, the data must be evaluated in a large group of patients, although several authors have reported the problem of adverse drug reactions and drug interactions in both elderly and polytreated patients [27,28,29]. This suggests the need for deprescribing. Another limitation is the unavailability of previous treatments, as well as of genetic data. Therefore, we are not able to say if the development of these symptoms could be related to genetic predisposition (e.g., polymorphism in liver cytochromes or in renal excretion pumps).

## 3. Conclusions

The present case report shows the need for wise prescription and deprescription in older people, trying to avoid all the potentially inappropriate drugs, and promoting the cholinergic approach for improving memory. Drug-induced symptoms are the second most frequent medical problem that the geriatrician encounters, and collaboration with a pharmacologist can help to optimize pharmacotherapy by deprescribing inappropriate agents.

## Figures and Tables

**Table 1:** Description of drugs administered to our patient. Inappropriate drugs are in bold (see explanation in the section discussion). Pantoprazole is reported in italics (see the section discussion).

Treatment	Name and Dosage	Indication
Apixaban	Eliquis 5 mg bid	Atrial fibrillation
Bisoprolol	Cardicor1.25 mg twice a day	Atrial fibrillation
Repaglinide	Novonorm 0.5 mg bid	Diabetes
Atorvastatin	Torvast 20 mg once a day	Hypercholesterolemia
Tiotropium bromide	Spiriva 18 μgrams 1 puff once a day	Chronic obstructive pulmonary disease
Ramipril/hydrochlorothiazide	Triatec HCT 5/25 mg once a day	Blood Hypertension
**Chlorpromazine**	**Largactil 25 mg bedtime**	**Psychosis**
**Risperidone**	**Risperdal 1 mg bedtime**	**Psychosis**
**Levodopa/Carbidopa**	**Sinemet 100/25 mg once a day**	**Parkinsonism**
*N*-demethyl-diazepam	En ten drops at bedtime	Anxiety
*Pantoprazole*	Pantopan 20 mg	Gastric protection

**Table 2:** Laboratory tests in patient at the time of the enrollment and of follow-ups (3 and 6 months).

	Admission	3 Months	6 Months	Normal Values
Blood Glucose	124	122	123	74–106 mg/dL
Glycated hemoglobin HbA_1c_	6.5	6.5	6.4	5.7–6.4%
Blood cholesterol	220	210	210	70–200 mg/dL
Cholesterol-HDL	45	44	44	35–39 mg/dL
Cholesterol-LDL	145	128	125	<130 mg/dL
Triglycerides	125	124	-	50–150 mg/dL
Aspartate amino transferase	32	-	-	5–50 IU/L
Alanine amino transferase	31	-	-	5–50 IU/L
Creatinine	92	-	-	0.7–1.2 mg/dL
White blood cells	7,850	-	-	4500–11,000 cells per µL
Red Cells	3.4	-	-	4.2–5.9 × 10^6^ cells/mcL
Folate	9	-	-	2.5–20 ng/mL
Vitamin B12	420	-	-	200–800 pg/mL
Homocysteine	11	-	-	<20 ng/mL
TSH	2.6	-	-	0.15–3.5 mU/L
FT3	4.5	-	-	3.0–8.0 pmol/L
FT4	1.2	-	-	0.9–2.4 ng/dL
C-reactive protein	8	-	-	0.5–10 mg/L
Vitamin D	12	25	30	30–60 ng/mL
Sodium	140	-	-	135–145 mM/L
Potassium	4.2	-	-	3.6–5.1 mM/L

**Table 3:** Neuropsychological tests performed on our patient during the study.

Mini-Mental State Examination (MMSE)	Admission	3 Months	6 Months	Comments
Orientation to time	2/5	**4/5**	4/5	**Improved at follow-up vs. admission**
Orientation to place	4/5	4/5	4/5	
Registration	3/3	3/3	3/3	
Attention and calculation	1/5	**2/5**	2/5	**Improved at follow-up vs. admission**
Recall	2/3	2/3	2/3	
Language	3/3	3/3	3/3	
Praxis	5/6	**6/6**	6/6	**Improved at follow-up vs. admission**
MMSE total score	20.4/30	**24.4/30**	24.4/30	**Normal value 26/30**
**MoCA (Montreal Cognitive Assessment)**				
Visuospatial abilities	3/5			
Naming	3/3			
Short-term memory recall	3/5			
Attention	3/6			
Language	2/3			
Abstract thinking	2/2			
Delayed recall	0/5			
Orientation to time and place	3/6			
MoCA total score	**19/30**			**Normal value 26/30**
				
**Clock Drawing Test (CDT)**	**6/10**			
Rey words	**21**			cut-off 28.53
				
**Stroop Test**				
Time	43			cut-off < 36.9 s
Mistakes	2			cut-off < 4.2
**TMT-A (Trail Making Test)**	normal range			
**TMT-B**	Unable			
**NPI scale (Neuropsychiatric Inventory Scale)**	27/144 episodes of anxiety; occasional visual hallucinations			
**GDS-15 items (Geriatric Depression Scale)**	8/15 points			cut-off ≥ 5 depressed mood
**Cumulative Insight Rating Scale (CIRS)**	0/8			
Activities of Daily Living (ADL)	2/6	**3/6**		**Improved at follow-up vs. admission**
Instrumental Activities of Daily Living (IADL)	1/5	**2/5**		**Improved at follow-up vs. admission**

**Table 4:** Inappropriate drugs according to the Beers criteria [11]. Drugs in bold were being taken by our patient.

• Digitalis (dosage/day > 0.125 mg/day)
• **Plasma long half-life BDZ** (diazepam, **N-demethyl-diazepam**, chlordiazepoxide)
• Tricyclic Antidepressants (amitriptyline, imipramine, chlorimipramine)
• **Phenotiazines with piperidine structure** (thioridazine, **chlorpromazine**)
• Butyrophenones (haloperidol)
• **Atypical AP** (clozapine, **risperidone**, olanzapine, quetiapine, aripiprazole)
• Semisynthetic belladonna alkaloids, quaternary ammonium compounds (butylscopolamine)
• Systemic antihistamine drugs (promethazine, diphenydramine)
• MethylDopa, Nifedipine, Disopyramide
• Adrenergic alpha1-blockers (doxazosin, terazosin)
• Presynaptic alpha2-agonists (clonidine)
• Laxatives (sodium picosulfate, bisacodyl)
• Antidiarrhea (loperamide)
• Prokinetics (metoclopramide)
• Class III antiarrythmics (amiodarone)
• NSAIDs (aspirin, indomethacin, naproxen, oxicams)
